# The Effects of Curcumin on Wound Healing in a Rat Model of Nasal Mucosal Trauma

**DOI:** 10.1155/2017/9452392

**Published:** 2017-09-05

**Authors:** Gokhan Emiroglu, Zerrin Ozergin Coskun, Yildiray Kalkan, Ozlem Celebi Erdivanli, Levent Tumkaya, Suat Terzi, Abdulkadir Özgür, Munir Demirci, Engin Dursun

**Affiliations:** ^1^Department of Otorhinolaryngology, Recep Tayyip Erdogan University Medical Faculty, Rize, Turkey; ^2^Department of Histology and Embryology, Recep Tayyip Erdogan University Medical Faculty, Rize, Turkey

## Abstract

We explored the effects of topical curcumin on the healing of nasal mucosal wounds. A total of 32 Sprague-Dawley Albino rats were randomized in equal numbers into four groups, and unilateral nasal wounds were created using an interdental brush. Group 1 (the sham-control group) contained untreated rats with traumatized right-side nasal cavities; Group 2 and 3 rats were similarly traumatized and treated with topical curcumin (5 and 10 mg/mL) dissolved in dimethyl sulfoxide daily for 7 days after trauma; Group 4 rats were treated with topical dimethyl sulfoxide only. All rats were decapitated on day 15 and the healing sites evaluated by blinded observers in terms of the presence of cellular hyperplasia, goblet cell hypertrophy and degeneration, leucocytic infiltration, ciliary loss and degeneration, edema, and vascular dilation. On histopathological evaluation, all of cellular hyperplasia, leukocytic infiltration, and edema were significantly reduced in Group 3 compared with Group 1 (*p* = 0.001, *p* = 0.004, and *p* = 0.008, resp.). Thus, curcumin reduced the inflammatory response and significantly accelerated wound healing.

## 1. Introduction 

*Curcuma longa* (turmeric) is a plant of the ginger family and has been used for many years in both India and China as both a spice and a traditional medicine. In India, turmeric has been widely used to treat biliary and hepatic diseases, coughs, diabetic ulcers, and rheumatic conditions. Curcumin is the most active form of the three different curcumoid extracts in the roots of plants [1]. Curcumin is insoluble in water but soluble in oil and ethanol. The chemical structure of curcumin (diferuloylmethane) was elucidated by Milobedska et al. in 1910. Curcumin has exerted antioxidant, antiaging, anti-inflammatory, immunomodulatory, wound healing, antitumoral, and antipsoriatic effects in various studies [1–3]. However, the effect of curcumin on nasal mucosal wounds has not yet been studied.

The nose is the first organ of the respiratory system, filtering, warming, and moistening inhaled air. The nose is also an olfactory organ and plays various roles in resonation, middle ear ventilation, drainage of the paranasal sinuses, sneezing, heat regulation, and the nasopulmonary reflexes. In such contexts, nasal mucosal health is key. The nasal respiratory mucosa is lined with ciliary, pseudostratified, columnar epithelial cells, and mucus-secreting goblet cells interspersed with ciliary cells [4].

Nasal mucosal wounding may develop after infection, accidents, and iatrogenically (septum/sinus surgeries). Wound healing after such injury is essential for maintenance of nasal ventilation and other functions. Wound healing is a complex process featuring a balance between inflammation, reepithelization, and matrix deposition; these processes are regulated by cytokines and growth factors released from leucocytes, fibroblasts, and epithelial cells [5]. Nasal mucosal trauma is associated with loss of goblet cells, thickening of the subepithelial layer (attributable to increased inflammation), neovascularization, and fibrosis. During healing, the numbers of goblet and ciliary cells, and the thickness of the subepithelial tissue, gradually approach those of healthy tissue [6].

In the present study, we histopathologically explored the effects of curcumin on wound healing of the nasal mucosa.

## 2. Materials and Methods 

The animal studies were approved by our Institutional Animal Care and Use Committee (approval number 2015-7).

### 2.1. Animals

We obtained 32 adult, male, 3–5-month-old Sprague-Dawley Albino (250–350 g) rats from our Institutional Animal Care Unit. After acclimation, the animals were divided into four groups and held in our experimental animal unit under a 12 h/12 h light/dark cycle at 55–60% humidity and 22 ± 3°C. Ad libitum food and water were provided. The animals were always treated as mandated ones by the Helsinki Declaration. Nasal mucosal trauma was inflicted under anesthesia induced by intraperitoneal injection of ketamine hydrochloride (Alfamine®, Alfasan International BV, Woerden, Holland) and xylazine HCl (Alfazyne®, Alfasan) (50 mg/kg and 10 mg/kg, resp.).

### 2.2. Study Design

**Figure 1:**
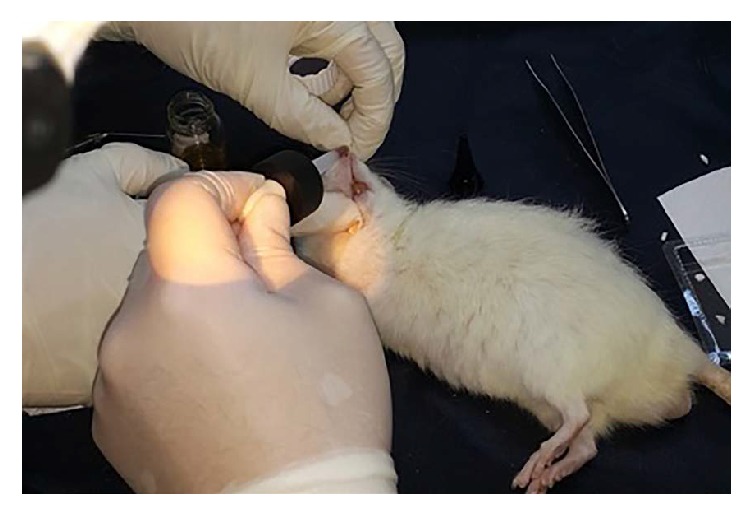
Application of drops to the right nasal cavity of the rat after traumatization.

Mechanical injury was inflicted by inserting an interdental brush (10 mm in width) through the right nostril [6]; an absorbable gelatin sponge (Gelfoam®; Pfizer Inc., New York, NY, USA) was then put in place (Figure 1). Group 1 (*n* = 8) was the sham-control group (no intervention). Groups 2 (*n* = 8) and 3 (*n* = 8) were the low- and high-curcumin groups, respectively. Rats in these groups received three drops daily of 5 mg/mL and 10 mg/mL solutions of curcumin (Sigma-Aldrich®, St. Louis, MO, USA; catalog number: C1386) in dimethyl sulfoxide (DMS) (Sigma-Aldrich; catalog number D8418), for 7 days. Group 4 (*n* = 8) was the DMS-only group. All rats were decapitated on day 15.

### 2.3. Histological Evaluation

Sections prepared from the traumatized regions of the nasal mucosa were stained with Masson's trichrome dye (which reveals wound healing better than does hematoxylin-and-eosin staining). The slides were viewed under 20x and 40x magnification employing a light microscope. For each animal, five slides were randomly selected from the 10 slides containing the traumatized areas. All of cellular hyperplasia, goblet cell hypertrophy and degeneration, leukocytic infiltration, ciliary loss and degeneration, edema, and vascular dilatation were evaluated by two histologists blinded to group status. The histopathological results were graded as mild (+), moderate (++), severe (+++), or very severe (++++).

### 2.4. Statistical Analysis

Data were analyzed using the IBM Statistical Package for the Social Sciences ver. 16 (SPSS Inc., Chicago, IL, USA). Nonparametric tests (the Mann–Whitney *U* test and the Kruskal-Wallis Test) were employed to compare normally distributed data. Continuous data are presented as means ± standard deviations or as medians [minima–maxima], as appropriate. Bonferroni post hoc analysis was applied when multiple comparisons were performed. The original significance level (*p* value) of 0.05 was adjusted to *p* < 0.0083 for individual comparisons when the Bonferroni correction was used.

## 3. Results 

**Figure 2:**
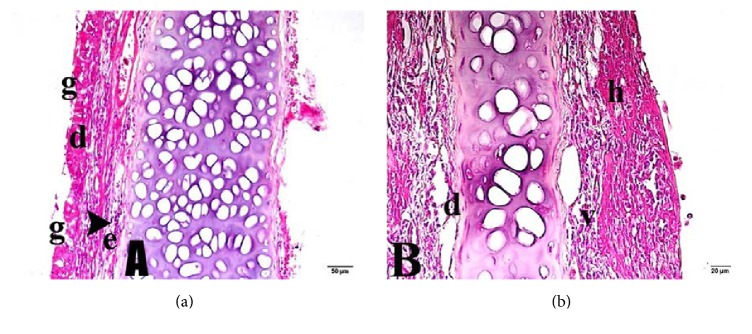
Nasal mucosa of Group 1 (sham-control group). (a) d: vascular dilatation; g: goblet cell; e: edema; arrowhead: leukocytic infiltration. (b) v: vacuolization; h: bleeding. Trichrome staining: (a) 20x and (b) 40x.

Group 1 (sham-control) rats exhibited goblet cell deformation, a reduction in goblet cell numbers, and mucus discharge from hypertrophic goblet cells (Figure 2). Vascular dilatation and perivascular edema were also evident. Pseudostratified columnar epithelial cells were absent from some areas. Diffuse ciliar desquamation of epithelial cells accompanied by areas of degeneration was observed; some cells both were pycnotic and had commenced apoptosis. Hyperplastic processes were evident, associated with patchy regions of epithelial condensation.

**Figure 3:**
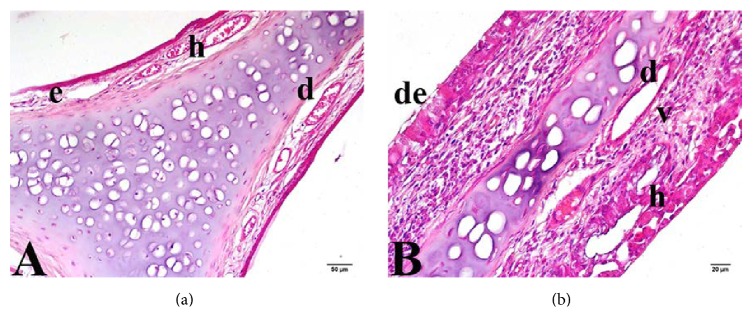
Nasal mucosa of Group 2 (low-dose curcumin group). (a) e: edema; h: bleeding; d: vascular dilatation. (b) h: bleeding; v: vacuolization; d: vascular dilatation; de: degenerative cell. Trichrome staining: (a) 20x and (b) 40x.

In Group 2 (the low-dose curcumin group), goblet cells were more numerous than in Group 1, but statistical significance was not attained (Table 1). The hypertrophic goblet cells lacked mucus. Compared with Group 1, the levels of epithelial cell desquamation and ciliary loss were reduced, and the extent of healing of degenerated areas increased, with borderline significance (*p* = 0.009). As in Group 1, some cells were pycnotic and some apoptotic. A few areas of vacuolization attributable to cell loss were observed (Figure 3). Groups 1 and 2 did not differ significantly in terms of hyperplasia, edema, vascular dilatation, or leukocytic infiltration (*p* > 0.008).

**Figure 4:**
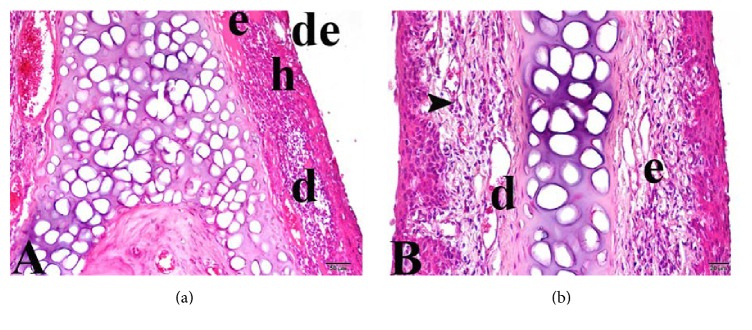
Nasal mucosa of Group 3 (high-dose curcumin group). (a) e: edema; h: bleeding; d: vascular dilatation; de: degenerative cell. (b) e: edema; d: vascular dilatation; arrowhead: leukocytic infiltration. Trichrome staining: (a) 20x and (b) 40x.

Compared with Group 1, Group 3 (the high-dose curcumin group) exhibited significant reductions in the extent of edematous mucosal areas, cellular hyperplasia, and leukocytic infiltration (*p* = 0.008, *p* = 0.001, and *p* = 0.004, resp.) (Figure 4), but the extent of vascular dilatation did not differ between the groups.

**Figure 5:**
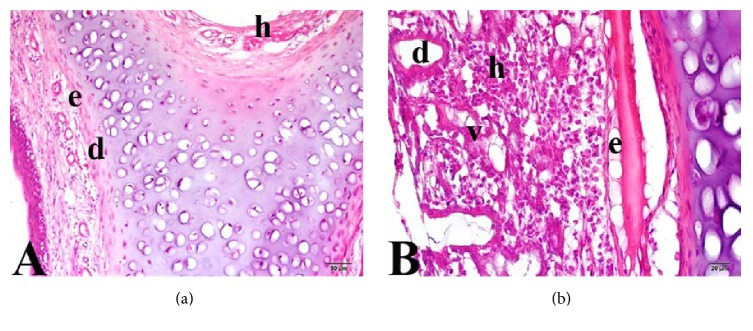
Nasal mucosa of Group 4 (DMS group). (a) e: edema; h: bleeding; d: dilatation. (b) e: edema; h: bleeding; v: vacuolization; d: vascular dilatation. Trichrome staining: (a) 20x and (b) 40x.

In Group 4 (the DMS group), the histopathological changes were similar to those of Group 1 (Figure 5). No significant between-group difference was evident in any of cellular hyperplasia, goblet cell hypertrophy or degeneration, leukocytic infiltration, ciliary loss or degeneration, edema, or vascular dilatation.

## 4. Discussion

Curcumin reduced the inflammatory response and accelerated wound healing in the nasal mucosa. Wound healing is a complex process with four interdependent phases: an early inflammatory phase (prominent within the first 2 days); a late inflammatory phase (commencing on day 2 or 3 after injury); a proliferative phase (days 4–21); and a remodeling phase (21 days to 1 year) [3, 7]. Edema and inflammation are associated with increases in vascular permeability, leukocytic infiltration, and extravasation of fluid from blood vessels to tissues. Wound repair is associated with normalization of vascular permeability, reduced leukocytic migration and edema, fibroblast proliferation, epithelial hyperplasia, enhanced regeneration, collagen deposition, and reduced ciliary cell loss and degeneration [6].

Lima et al. histopathologically studied the effects of trauma on the nasal mucosa; rats were sacrificed at 1 hour and 2, 5, 14, and 28 days, after trauma. On light microscopy, significant increases were evident by day 2 in both wound-edge edema and the subepithelial thickness index. By day 5, reepithelization had commenced, characterized by the presence of undifferentiated epithelial cells and an edematous lamina propria wherein monocytes had replaced neutrophils. Although goblet cell numbers had increased by day 14, irregularities in both goblet and ciliary cells, and epithelial layer thickening, persisted. By day 28, the respiratory mucosa had regained its normal structural anatomy [3].

Although the effects of curcumin on various tissues have been studied, its effect on wound healing of the nasal mucosa has not been examined to date. Both Groups 2 and 3 exhibited significant cellular regeneration and reductions in cell loss from the respiratory epithelial layer and near-significant reductions in ciliary loss and degeneration (*p* = 0.009; *p* = 0.01).

Topical curcumin (10 mg/mL) significantly reduced edema, cellular hyperplasia, and leukocytic infiltration compared with the sham-control group (*p* = 0.008, *p* = 0.001, and *p* = 0.004, resp.). However, such changes were not observed in Group 2 (the 5 mg/mL curcumin group). Thus, high-dose topical curcumin is required to exert an anti-inflammatory effect. Treatment with 10 mg/mL curcumin reduced cellular hyperplasia to the normal level and accelerated wound healing (Table 2).

Curcumin exerts anti-inflammatory, anti-infectious, and antioxidant effects [8–11], inhibiting the production of two cytokines (IL-1 and TNF-*α*) that activate the monocytes and macrophages playing important roles in regulation of the inflammatory response [12]. Curcumin inhibits the action of transcription factor NF-(*κ*)B (nuclear factor kappa-light-chain-enhancer of activated B cells) [13]. One of the primary physiological roles of nuclear factor, NF-(*κ*)B, is in the immune system. In particular, NF-(*κ*)B family members control the transcription of cytokines that regulate cellular differentiation, survival, and proliferation. In addition, NF-(*κ*)B also contributes to the development and survival of the cells and tissues that carry out immune responses in mammals [14].

Joe et al. showed that application of curcumin-loaded polymeric bandages to rats significantly reduced antioxidant enzyme expression, attributable to the fact that curcumin reduced lipid peroxidation and thus lowered the requirement for antioxidant enzymes [13]. The level of high-aldehyde collagen also increased. Panchatcharam et al. observed both an increase in collagen level and earlier maturation of collagen fibers [8].

In a diabetic rat model, topical application of curcumin to wounds improved the organization of granulation tissue and was associated with higher numbers of myofibroblasts and smaller capillaries [15]. However, in our study, curcumin did not reduce vascular dilatation; curcumin may promote both neovascularization and small capillary formation.

Curcumin exerts an apoptotic effect early in wound healing. In rats bearing curcumin-containing bandages, early cell death (DNA fragmentation) was apparent 4 days after treatment [13]. Akbik et al. showed that, during wound healing, curcumin prolonged the inflammatory phase only minimally and accelerated the proliferation phase [1], which generally commences 4 to 21 days after injury. However, the phases of wound healing are often superimposed. Reepithelization (part of the proliferative phase) probably commences immediately after trauma [3].

Joe et al. showed that, in a control group, apoptosis increased 11 days after injury, but all early apoptotic cells had been eliminated in the curcumin-treated group by this time, showing that the untreated group was in the early phase of wound healing but the curcumin-treated group was in the proliferative phase [13].

In the rat, curcumin facilitated complete wound reepithelization, reducing the epithelization period from 23 to 11 days compared with a control group [12]. In our study, 15 days of high-dose curcumin treatment was associated with reduced ciliary degeneration and loss and less cellular hyperplasia, compared with the sham-control group, and the respiratory epithelium became normalized. Thus, curcumin accelerated epithelization of the wound site.

Curcumin is insoluble in water but soluble in oil and alcohol. Thus, curcumin is poorly absorbed and is present in serum only in a compound form. Curcumin is extensively first-pass metabolized and is sensitive to light [16]. Many studies have shown that topical application of curcumin to wound sites was more effective than oral intake, affording higher local concentrations [16, 17].

DMS (used to dissolve curcumin) is a valuable organic sulfurous solvent derived from wood. DMS is transported from the skin or even the nails into the body and enhances the absorption of dissolved materials [18, 19]. We used a curcumin/DMS solution for topical application; DMS alone did not affect wound site healing. Some studies have evaluated curcumin nanoformulations. Such curcumin preparations (small particles of high surface area) exhibited enhanced intracellular penetration [20, 21]. The nanoparticles penetrated cancer cells to a greater extent than did simpler preparations and reduced cell proliferation [20]. Also, nanoformulated curcumin exhibits good bioavailability and a long half-life and can be effectively dispersed in water [21]. Thus, aqueous curcumin creams may be prepared.

A limitation of our study is the lack of electron microscopic histopathological data (our institutional resources are limited). However, significant healing improvements were readily evident under a light microscope. Curcumin notably accelerated wound healing and can thus be used to treat nasal mucosal injuries persisting after nasal trauma, septoplasty, turbinate surgery, functional endoscopic sinus surgery, and tumor removal. The optimal dose level and interval, and the maximum tolerated dose, remain to be determined. Also, alternative delivery mechanisms deserve attention.

## Figures and Tables

**Table 1:** The histopathological data (^+^*p* = 0.001; ^*∗*^*p* = 0.004; ^#^*p* = 0.008).

Group	Cellular hyperplasia	Goblet cell hypertrophy-degeneration	Leucocyte infiltration	Loss of cilia and ciliary cell degeneration	Edema	Vascular dilatation
Group 1	3.00 ± 0.46^+^	3.00 ± 0.46	4.00 ± 0.35^*∗*^	4.00 ± 0.00	3.00 ± 0.35^≠^	3.00 ± 0.35
Group 2	3.00 ± 0.52	4.00 ± 0.52	4.00 ± 0.52	3.00 ± 0.46	3.50 ± 0.74	3.00 ± 0.64
Group 3	2.00 ± 0.35^+^	3.00 ± 0.53	3.00 ± 0.53^*∗*^	3.00 ± 0.71	2.00 ± 0.52^≠^	3.00 ± 0.53
Group 4	3.00 ± 0.35	3.00 ± 0.53	4.00 ± 0.35	4.00 ± 0.00	3.00 ± 0.46	3.00 ± 0.53

**Table 2:** Histopathological grades.

Group	Cellular hyperplasia	Goblet cell hypertrophy/degeneration	Leucocyte infiltration	Loss of cilia and ciliary degeneration	Edema	Vascular dilatation
1	**+++**	**+++**	**++++**	**++++**	**+++**	**+++**
2	**++/+++**	**+++/++++**	**+++/++++**	**+++/++++**	**+++**/**++++**	**+++**
3	**++**	**+++**	**+++**	**+++/++++**	**++/+++**	**+++**
4	**+++**	**+++**	**++++**	**++++**	**+++**	**+++**

+: mild, ++: moderate, +++: severe, and ++++: very severe.
